# Safeguarding the patient: a grounded theory study of registered nurse anesthetists’ main concerns in the process of extubation in the anesthesia setting

**DOI:** 10.1186/s12912-022-00817-1

**Published:** 2022-03-09

**Authors:** Linda Rönnberg, Christina Melin-Johansson, Ove Hellzén, Ulrica Nilsson, Marie Häggström

**Affiliations:** 1 Department of Nursing Sciences, Mid Sweden University, Östersund, Sweden; 2 Department of Health Care Sciences, Ersta Sköndal Bräcke University college, Stockholm, Sweden; 3 Department of Nursing Sciences, Mid Sweden University, Sundsvall, Sweden; 4 Department of Neurobiology, Care Sciences and Society, Karolinska Institutet, Stockholm, Sweden; 5 Perioperative Medicine and Intensive Care, Karolinska University Hospital, Stockholm, Sweden

**Keywords:** Anesthesia, Anesthetic nursing, Extubation, Grounded theory, Registered nurse anesthetist, Safeguarding

## Abstract

**Background:**

The process of extubation is complex as it takes place in the technical and challenging environment of the operating room. The extubation is related to complications of varying severity and a critical moment for the patient, who is in a vulnerable condition when emerging from anesthesia. Registered Nurse Anesthetists (RNAs) in Sweden have specialist training and performs extubations independently or in collaboration with an anesthesiologist.

**Aim:**

To obtain a deeper understanding of Registered Nurse Anesthetists’ main concerns and how they resolve these in the process of extubation when caring for a patient during general anesthesia.

**Participants:**

A total of 17 RNAs, eight male and nine female, were included in the study. Twelve RNAs in the first step of data collection (I); and five RNAs the second step of data collection (II).

**Method:**

A classic grounded theory approach with a qualitative design was used for this study.

**Findings:**

The RNAs’ main concern in the process of extubation were *Safeguarding the patient in a highly technological environment*, which the solved by *Maintaining adaptability*. Facilitators as well as challenges affected how the RNAs solved their main concern and represented the categories: ‘Having a back-up plan’, ‘Getting into the right frame of mind’, ‘Evaluating the patient’s reactions’, ‘Using one’s own experience’, ‘Dealing with uncertainty’, ‘Pressure from others’, and ‘Being interrupted’. The theory, *Safeguarding the patient in the process of extubation*, emerged.

**Conclusion:**

To be able to safeguard the patient in a highly technological environment, the RNAs must oscillate between facilitators and challenges. By maintaining adaptability, the RNAs resolved the difficulties of oscillating, indicating a need for finding a balance between maintaining attentiveness on what is important to keep the patient safe in the process of extubation and all of the disturbances present in the OR.

## Contribution to the literature

What is already known about the topic?

- Registered Nurse Anesthetists (RNA) combine theoretical knowledge with clinical experience and intuition when deciding when to extubate
- RNAs experience loneliness, lack of respect and lack of guidance in the process of extubation
- During anesthetic care the RNAs establish a relationship with the patient

What this paper adds?

- Illuminates the different layers of actions that the RNAs utilize in the process of extubation
- Explains the lifelong learning involved in becoming an RNA and in developing capabilities to safeguard patients during the process of extubation
- Clarifies how important it is that RNAs must take great consideration to be able to focus in this critical moment
- Shows the importance of supporting those with less working experience

## Introduction

The operating room (OR) is a complex, highly technological space, presenting many challenges for the professionals in the surgical team, who work around the patient [1] within a stressful working environment [2]. The surgical team in Sweden usually consists of an anesthesiologist, a Registered Nurse Anesthetist (RNA), a surgeon, an operating theatre nurse and other nursing staff, and often students in training. Team membership can vary over time and may not consist of the same persons throughout any single anesthesia [1]. The composition of the anesthesia team differs between countries, but, in Sweden, consists of one anesthesiologist, who has medical responsibility, and one or more RNAs [3]. The RNAs in Sweden are Registered Nurses who have acquired a one-year post-registration qualification for anesthesia care [4, 5]. The RNAs have an independent responsibility for the anesthesiological nursing care of patients in a high-tech environment, where having specific knowledge and experience are essential. For patients who have a physical status of American Society of Anesthesiologists (ASA) class I or II, the RNAs independently induce, complete and carry out general anesthesia (GA) according to specified protocols and under the supervision of an anesthesiologist [6]. For patients who have a physical status of III or higher, or for all patients undergoing acute surgery, RNAs plan and administer GA in collaboration with an anesthesiologist [7]. In the anesthesia setting, the RNAs share the responsibility of anesthesia care with anesthesiologists [7]. Patients undergoing GA often must be intubated with an endotracheal tube to ensure a secure airway and adequate breathing. Intubation is necessary if the patient is at risk of aspiration of secretion, blood or stomach content, in need of neuromuscular relaxation, or is critically ill [8]. In Sweden, responsibility for performing the extubation is a shared concern; between the RNAs and anesthesiologists. Usually, the plan is to perform the extubation of the endotracheal tube after GA in the OR [9]. At the extubation, the patient moves from a controlled phase, having an established airway, to an uncontrolled situation, after the extubation, without the endotracheal tube to secure the airway and provide adequate breathing [10]. At the point of emergence from anesthesia, the patient is in a vulnerable situation and the extubation itself is related to a risk of causing complications of varying severity [11].

When caring for a patient in the process of extubation, the RNAs perform their care in a technical and challenging environment. Although the work environment is highly technical, the decision-making practices at the extubation do not relate only to the technique of performing the extubation. Nor has it been shown to relate only to a single moment at the end of anesthesia. In our earlier studies with RNAs’ and Anesthesiologists’ experiences, it emerged that the extubation is a process [12, 13]. This process includes seeing beyond the monitors, combining previous experience with being a step ahead, and using intuition when making the decision on when to perform the extubation – a decision that is also based on neuromuscular monitoring and assessment of anaesthesia depth. We did learn much about the RNAs’ process of extubation from these studies, but more knowledge is needed to identify the RNAs’ approach within the anesthesia setting. To our knowledge, there is a lack of research regarding how the RNAs act and reflect in the process of extubation, and very little that focuses on their main concerns in a process that is described as being complex and critical for the patient.

### Aim

To obtain a deeper understanding of Registered Nurse Anesthetists’ main concerns and how they resolve these in the process of extubation when caring for a patient during general anesthesia.

## Methods

A classic grounded theory (GT) approach with a qualitative design was used for this study [14]. By focusing on the RNAs and their perceived problems or main concerns in the process of extubation, the purpose was to understand the actions and behaviors of those involved in this process from their perspective [15].

### Participants and procedure

Three hospitals in Sweden, of different sizes and various geographical locations, were invited to take part in the study. After being given permission to conduct the study from the head of the anesthesia departments, all RNAs employed in these departments were informed about the study, in person and in writing, and were invited to participate. A total of 17 RNAs, eight male and nine female, agreed to participate and were included in the study: twelve RNAs (six male and six female) were recruited via consecutive sampling from two hospitals (seven from one university hospital (A), and five from one county hospital (B)) and participated in the first step of data collection (I); and five RNAs were recruited from another county hospital (C), in a theoretical sample, to participate in the second step of data collection (II). All RNAs who agreed to participate completed an informed consent form.

### Data collection

In the initial phase of data collection (data collection I), data were collected from the RNAs using individual interviews. These comprised reflective interviews with open questions, focusing on the observations and reflections made by the RNAs on their main concerns in the process of extubation. Thus, after an initial analysis, more data were gathered in order to continue the constant comparison [16]. In data collection II, questions revealed in the initial analysis regarding the core category were used to gather more data until saturation was reached.

In data collection I and II, interviews were audio-recorded and transcribed verbatim by the first author. All identifying information was deleted and replaced with ‘XX’ in the transcripts. Memos relating to ideas about evolving codes and the relationships between them, and about the problem or main concern the participants talked about and how they deal with these in practice, were written after each interview. These memos were used to document ideas about the codes and the relationships between them [16].

#### Data collection I

Data were initially collected from RNAs from one university hospital (A) and one county hospital (B). All RNAs who were on duty on the day that the observations took place and who had completed an informed consent form were able to be included in the study. The initial observations of the RNAs, focusing on the process of extubation, were video-recorded. Afterwards, reflective individual interviews were performed and audio-recorded. The video recording started when the patient arrived at the OR and ended when the patient left the room. This period of time for the video-recording was chosen because the extubation process has been described to already begin at the start of anesthesia in earlier studies [12, 13]. The video camera was placed on a tripod in the OR and set so that only the patient was visible from the shoulders up on the recording. In both hospital A and B, the hospital photographer set up the camera, and the recordings were made by the first author. The patient’s head was covered with a cap or towel to protect their anonymity, the extubation was clearly visible in all audio-visual-recordings. The recording was used as a tool to allow the RNAs to reflect upon the process of extubation during the interviews, thus the reflective interviews lasted from 75 to 155 min (the duration of the audio-visual-recording), or a few minutes longer, where participants made some further reflections after the recordings had ended. Only the first author and the observed RNA viewed the respective audio-visual-recording. Directly after the interviews, the video file was erased to protect the patient’s identity and the RNA’s anonymity. The first author followed and observed the RNAs throughout the entire anesthesia period, from the moment they started to prepare the patient, to meeting the patient in the preoperative unit, until they handed the patient over to the postoperative unit. The first author acted as a participant observer, taking fieldnotes and observing the RNA during the process of extubation. The fieldnotes were used to guide the interviews, and to clarify any queries raised by the observer relating the extubations and also to contribute to the codes generated in the analysis of the data. The fieldnotes were written by hand on A4 paper and consisted of one-to-two sheets of notes for each observation. The interviews were conducted while watching the audio-visual-recordings and adopted an open approach, asking the RNAs to reflect on any concerns they had during the process of extubation. The first question asked was: “Please reflect upon your concerns regarding this extubation”. Questions such as: “Can you tell me more?”, “What did you think when … happened?”, or “How did you handle that?” were asked.

#### Data collection II

In the second step of data collection (II), a theoretical sample was recruited, with the purpose of following clues and leads arising when analyzing the initial data (I) [17]. Five RNAs were recruited from another county hospital (C) after completing an informed consent form. These participants were chosen for their theoretical relevance and for further development of the categories and the main concerns identified in the initial analysis [14] and asked to participate in in-depth individual interviews. Due to the Covid-19 pandemic, these were performed using the video conferencing suite, Zoom [18]. The interviews lasted from 15 to 30 min, were digitally recorded, and transcribed verbatim by the first author. The questions were designed to seek additional information to reach saturation in the development of categories, provide insight in what might be missing, and to highlight gaps in the data to further develop the analysis and creation of categories and main concerns [19]. Questions such as: “What’s important for you to be able to safeguard the patient in the process of extubation?”, and “Can you reflect upon ‘taking the safe path’ regarding the process of extubation?” were asked.

### Data analysis

**Fig. 1:**
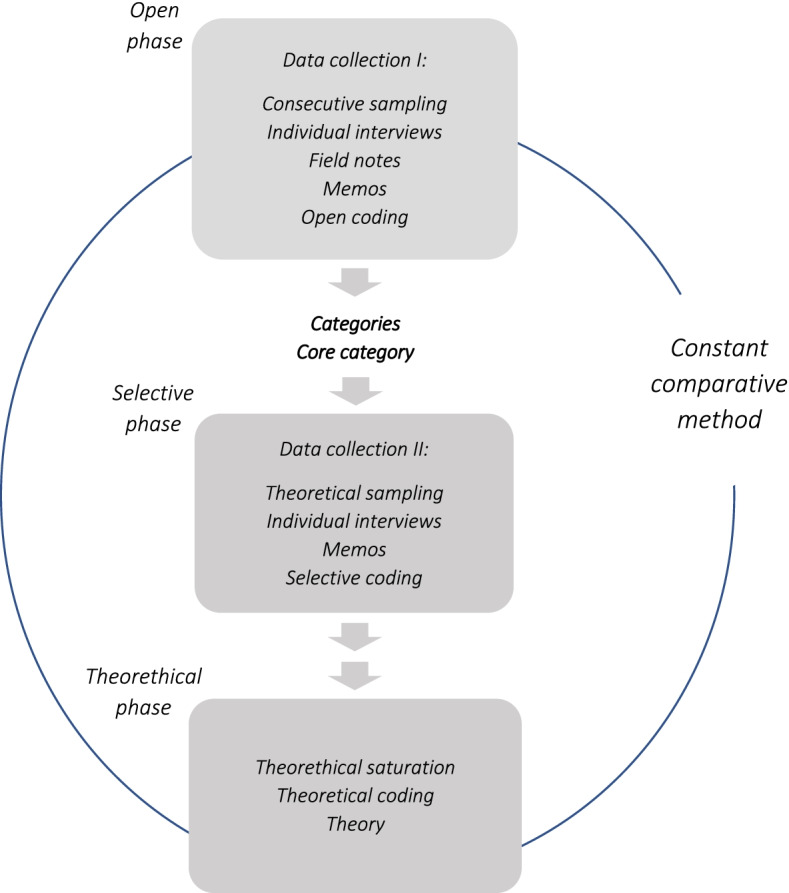
The research process of GT. In the first open phase data is coded and analysed open, categories are created and represent phenomena of importance for the participants. When the core category is decided and represent the category who solve the participants’ main concern, the next phase begins. In the selective phase, only the categories related to the core category remains and guides further data collection. When theoretical saturation is reached and the theoretical codes explains the relationships between the categories, the theory is generated. Through the process a constant comparative method is conducted [15, 20]

GT is a constant comparative method; data from one interview were compared with those from the others [14]. Following classic GT, the first stage of this comparison is the open coding process, where data were initially allocated a label based on their characteristics associated with the RNAs’ main concerns or how to solve these, see Fig. 1. In this initial inductive step in the coding process, where gathered data were broken down into smaller segments or words/phrases, repeating labels were then given a code representing latent patterns in the data [14].

The additional data collected from the theoretical sample in data collection II was analyzed in order to explain concepts and codes and to discover categories and the connections between them [17]. In this phase, a selective coding process was performed and the developing categories were formed around core concepts and the core category, “Maintaining adaptability” emerged. During this phase of analysis, only data related to the core category were included. In the next step, the theoretical coding stage, how the categories belonged together was determined by looking for patterns and forming hypothetical relationships between them [17]. Theoretical codes or code families were identified, for example, causes, interactions, and consequences [14]. This constant comparative analysis continued until the content of one source was compared to the content in all other sources of data [21]. When no new concepts emerged in the analysis, theoretical saturation was achieved [22].

During the analysis phase, as when collecting data, and when coding data, memos were written in order to capture and preserve ideas that encouraged the researcher to describe patterns in the data, and to reflect upon these as well as the relationship between categories [14].

### Ethical considerations

This study was conducted within the standards set out by the declaration of Helsinki [23] Prior to taking part, with the aim to minimize the impact the study may have on the participants’ physical and mental integrity and personality all participants received information, orally and in written form. The information underlined that their participation was voluntary, that the data would be treated with confidentiality, and that they could withdraw from the study at any time until the point at which the data had been analyzed. Before the audio-video-recorded observations took place, each participating RNA and patient signed an informed consent form. All other personnel in the OR were given verbal information about the study and were informed that the observations would be audio-visually recorded and that the recordings would be erased directly after the follow-up interview with the RNAs. During the observations, to inform all non-participants about the recording, a note was placed on the entrance to the OR, reading “ongoing video-recorded observation”. Due to the Covid-19 pandemic, the interviews in data collection II took place after working hours, so as not to take time away from patient care.

To ensure the quality of the research and that no data were lost, the theoretical constructions generated from the data, in an ongoing process, were checked against the participants’ own words to determine whether their meanings were relevant to the emerging theory [24, 17].

This study was given ethics approval by the regional ethical board in Umeå (Dnr 2014-19-31 M).

## Findings

### Safeguarding the patient in the process of extubation

**Fig. 2:**
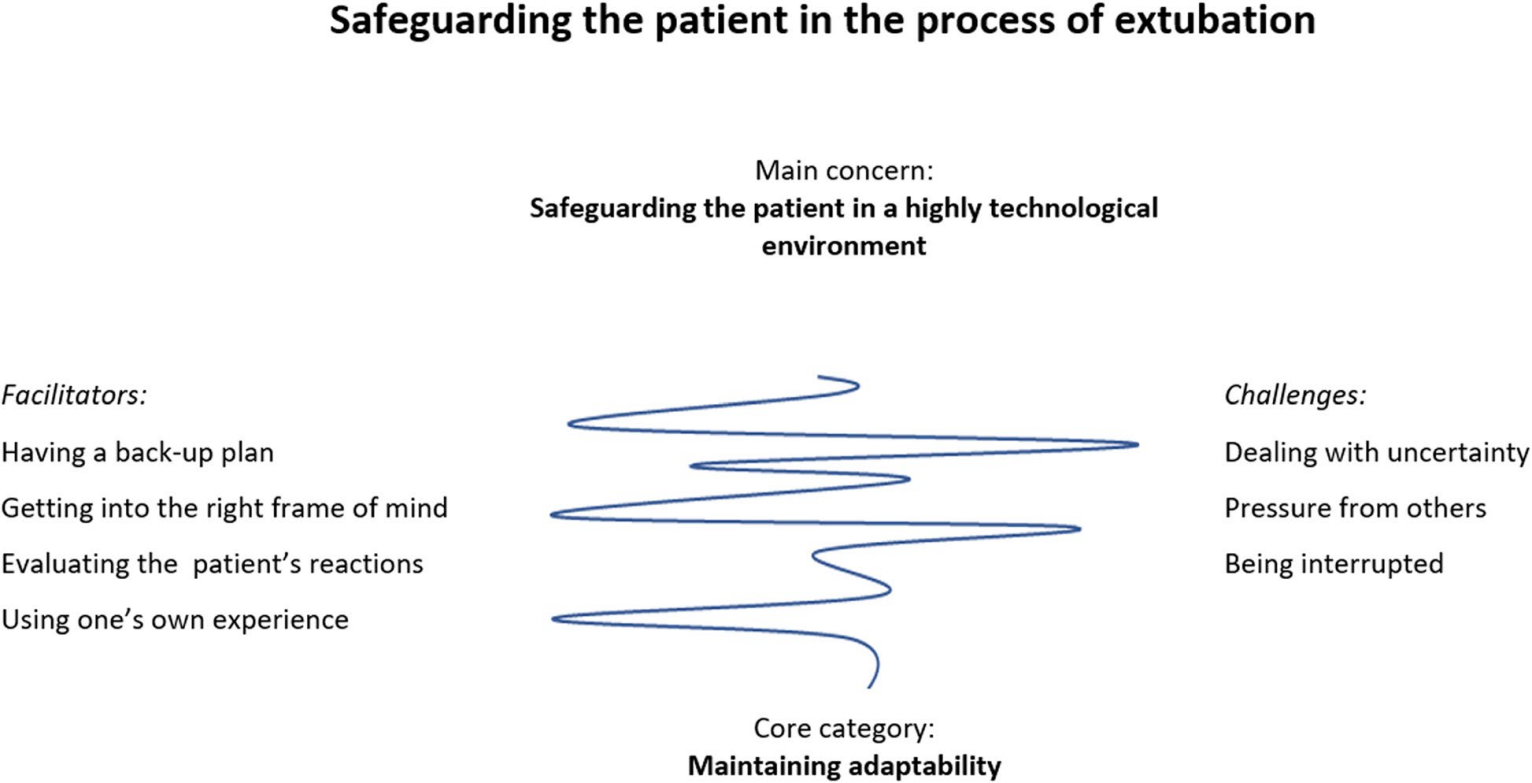
In the process of extubation, the RNAs’ main concerns are *Safeguarding the patient in a highly technological environment*, which they resolved as described by the core category *Maintaining adaptability*. The categories are divided into facilitators and challenges, between which the RNAs oscillate, and the grounded theory is *Safeguarding the patient in the process of extubation*

The generated grounded theory of this study was revealed to be *Safeguarding the patient in the process of extubation*. It was a matter of course to want to protect the patient, but the RNAs’ ability to do so in the complex environment and at the critical moment of the extubation was affected by elements that acted as both facilitators and challenges, headings in which the emerging categories are presented. The main concern of the RNAs, *Safeguarding the patient in a high technological environment,* was described by the RNAs as being resolved by *Maintaining adaptability*, representing the core category (see Fig. 2).

#### The main concern – safeguarding the patient in a highly technological environment

In *Safeguarding the patient in a highly technological environment*, the RNAs moved from being able to fully concentrate on safeguarding the patient in the process of extubation, to the reality of not being able to keep this focus in the highly technological environment of the OR. Distractions in the form of disturbances and interruptions from technical equipment and from other professionals, and their own vulnerability in being solely responsible for the safety of the patient, affected their focus. Their ability to safeguard the patient was also affected by the RNAs’ own limitations, such as being inexperienced or feeling insecure. This continual shift between concentration and distraction was explained as being problematic due to its unpredictability, because, as they explained, even if they had previous experience of patients reacting similarly to other patients undergoing surgical procedures or anesthetic agents, each patient and each extubation was unique. Also, the closer they came to the extubation, the more focused they needed to be, which was explained as being even harder to cope with for the inexperienced RNAs, due to the complexity of knowing when the surgical procedure is ending and feeling safe about standing up for their decision on when to extubate.

#### The core category – ´maintaining adaptability´

The core category, *Maintaining adaptability*, explained how the RNAs resolved their main concerns. To resolve this they maintained adaptability, and, in that way, indicated how they oscillated^1^ between being attentive and being distracted by disturbances in the process of extubation while still having to hold the line to safeguard the patient. This they achieved by building up a mental plan, staying focused and near the patient, interacting with them, and using their clinical experience, thereby putting patient safety first in the process of extubation. The RNAs moved between being focused and not being able to keep this focus due to disturbances. To be able to solve their main concerns, the RNAs explained that there were facilitators as well as challenges for this, as illustrated in Fig. 1. Maintaining adaptability included how the RNAs adapted their actions and managed changes in the patient’s condition, their ability to read and react to signals or reactions from the patient, and those from other professionals in the OR.

Possibilities to resolve their main concern of safeguarding the patient in a highly technological environment were affected by both facilitators and challenges and were indicated to differ between patients, RNAs, and each extubation. In this it was indicated that they acted as the patient’s advocate, remained vigilant, considered, reflected, and made decisions based on clinical judgement. But it was also explained that there is a constant shift towards losing this attentive focus due to disturbances in the OR. How much they oscillated depended on their own experience and ability to stay focused, even if the other professionals in the OR did not. One RNA shared, “The thing is, you need to know the art of staying focused even though they [the other professionals] draw your attention, knowing when you cannot leave the patient’s head for a second” (p. 3). Another said, “Nowadays I know if I dare to leave for a while to answer the phone or pick up a bandage, I did not know then, as a new RNA, to trust my own decision when it came to the extubation” (p. 13). It was also explained to be a matter of getting to know each patient’s reactions during anesthesia. It was the RNAs’ understanding of these responses that guided them, thereby allowing them to safeguard the patients. However, if they lost focus, they also missed out on these responses. Based on the RNAs’ experience and all the information they collected, a mental map was created, which they explained was used as a guide and helped them to stay attentive in the process of extubation.

#### Facilitators

In the process of extubation, the RNAs’ ability to safeguard the patient were explained to be facilitated by the categories, ‘Having a back-up plan’, ‘Getting into the right frame of mind’, ‘Evaluating the patient’s reactions’, and ‘Using one’s own experience’.

### Having a back-up plan

To be able to maintain adaptability to safeguard the patient was an enabler for the RNAs to plan for having someone else in the anesthesia team to call for assistance if an acute event occurred, and with whom they could share their plan for the extubation and feel supported by. “When feeling calm and secure myself, then I sort of transmit this to the patient or in my actions” (p. 8). To be given the opportunity to focus on the extubation and feeling supported facilitated them to safeguard the patient. “When being new here I always had someone to cover my back, when it came to the extubation” (p. 10). It was indicated that, to safeguard the patient, it was a facilitator to prepare a back-up plan, for example, to have another RNA in the OR together with them at the time of the extubation, even if they did not share the plan for the extubation with them, but expressed in the words, “needing some helping hands”.

### Getting into the right frame of mind

Another facilitator for safeguarding the patient by maintaining adaptability was explained by the RNAs as getting into the right frame of mind. Being the one meeting the patient before anesthesia, reading the patient’s medical records, and gathering information were all explained by the RNAs as being part of setting their frame of mind and enabling them to safeguard the patient in the process of extubation as well as to “start the safe path to the extubation” (p. 6). By being the one who stays by the patient’s side throughout the entire anesthesia, the RNAs were able to gather information and through that they felt calm. The RNAs explained that adopting this frame of mind enabled them to stay focused and maintain adaptability. The RNAs explained that, to safeguard the patient, they constantly assessed and had to make decisions; if it was difficult to ventilate or intubate, they knew that there would likely be a higher risk of complications, even at the extubation. “I know what to expect, so to speak, I know if it was difficult then I need to be on my toes at the ending as well” (p. 7). If they were there to observe how the patient reacted during the intubation or to the surgical procedures, the RNAs could be prepared for how the patient might react and were in the right frame of mind for the extubation.

### Evaluating the patients’ reactions

The RNAs’ safeguarding practices were guided by them evaluating the patient’s reactions to various stimuli and anesthetics. To be able to adjust their actions, it was important to maintain their adaptability. This was indicated to be an interaction between them and the patient; to be able to interact attentively, the RNAs needed to be focused on the patient: “Not only physically present, but mentally there for them” (p. 16). In the OR, the RNAs explained their interactions with the patient were invaluable for safeguarding them. They allowed the patient’s reactions to surgery, drugs or other stimuli guide them in making the decision on when to extubate: “When I saw her reaction then I knew, how she responded to that stimuli guided my choice of how to manage the extubation, it is a teamwork between us two” (p. 5). Through the process of extubation, they continuously evaluated how the patient responded to their actions, and, if the reactions were not what was expected, they then revised their actions. A further re-evaluation was then performed, acted upon, and evaluated. It was explained that the more focused they were, the easier it was to maintain attentiveness and safeguard the patient.

### Using one’s own experience

RNAs’ own experiences enhanced their ability to maintain adaptability when safeguarding the patient: “I’m formed by it, definitely, and it is the base for my decision. All the things I have seen, in the extubation I recognize and recall” (p. 9). Using their experience and good clinical judgement was indication that the RNAs were making wise decisions. Each extubation became a new experience to learn from and, thereby, they were facilitated to safeguard the patient. The RNAs indicated that, in having prior experience, they also have the courage to tell the others in the OR to be quiet when it was time for the extubation. “Today it is not a problem, really, I know what to do if they [other professionals] are loud or talk a lot” (p. 10). This was explained as a wise decision due to their knowledge of complications at the extubation because of noise in the OR; therefore, they instead took the opportunity to remind the others to keep quiet.

#### Challenges

When trying to maintain adaptability and solve the main concerns, the categories, ‘Dealing with uncertainty’, ‘Pressure from others’, and ‘Being Interrupted’ were explained to be challenges for solving the main concern.

### Dealing with uncertainty

When standing alone, facing the decision on when to extubate, perhaps not having shared the plan for the extubation with anyone, dealing with uncertainty was a challenge for the RNAs, making it difficult to safeguard the patient. It also made them feel vulnerable and lose focus, and this affected them in their ability to maintain adaptability. Not being familiar with the different expressions and cultures present in the OR, or when the RNAs were uncertain about when the operation was approaching its end, were explained as challenges that affected them. “When being new you didn’t know when to start to prepare for the ending, and you really hadn’t any clue of what to expect the first few times you removed a tube, because it comes with experience and practice” (p. 5). This also signified that the RNAs did not always have any alternative plan if complications occurred at the extubation: “When being new you don’t have the experience nor the competence to predict all the things that can happen if you extubate at the wrong time” (p. 17). The RNAs also explained that when they lacked experience they also lacked the feeling of knowing when to extubate, and with that came feelings of uncertainty. This was in contrast to those RNAs with more experience, who used their gut feelings when making the decision on when to extubate.

### Pressure from others

Pressure from others was another challenge, and maintaining adaptability was difficult in these moments. The RNAs explained that there was a risk of trying to speed up the extubation, due to comments and pressure from other professionals in the OR. These comments were indicated to arise due to time pressures or a lack of understanding that the patient is in a vulnerable condition at the extubation. This affected the RNAs in certain ways; they told of losing focus, not trusting their own decision, and their adaptability being affected in the extubation process: “If I know we have short of time then I start, you know, like touching and talking to the patient earlier and perhaps even removing it (the tube) earlier also, I know I shouldn’t, but I feel pressured” (p. 14). It was also explained how other professionals sometimes tried to wake the patient, by talking to them, or touching or moving the patient’s body, without asking the RNAs whether the time was appropriate. This left the RNAs in a difficult situation, which was explained to be a challenge for the RNAs, in trying to maintain adaptability and safeguard the patient while at the same time being distracted and feeling pressured to be effective and prepared for the next patient. Although RNAs were rarely left alone with the patient in the OR, other professionals were cleaning the room and preparing for the next patient, meaning that their focus was not on the patient and the RNA. This added to the RNAs’ feelings of being alone, especially when the extubation was delayed: “It’s hard to be the one taking a lot of time and being the reason for a delay, and not even being able to explain why I didn’t remove the tube earlier” (p. 8). This may increase the risk of complications occurring for the patient at the extubation.

### Being interrupted

In planning and preparing for the extubation, the RNAs explained that they are often interrupted for a variety of reasons, for example, phone calls, assisting the operating team staff with their sterile dress for surgery, or by being asked to fetch equipment from non-sterile areas. This places high demands on the RNAs’ ability to maintain adaptability. These interruptions were often made by other professionals in the team around the patient: “I had to leave the patient to answer the phone and then to get something to the theatre nurse, it was fine this time but sometimes it feels like they don’t respect me just staying bedside the patient” (p. 1).

When frequently interrupted, the RNAs indicated a loss of focus on their anesthesia duties and the plan that they had prepared for the extubation might need to be altered, thereby not safeguarding the patient. Often, the RNAs felt lonely with the decision on when to extubate, and they also indicated that they rarely shared the plan for the extubation with someone else if the patient was not a small child, or if there had been airway trouble at the intubation, or if acute events had occurred. Although knowing that it was an advantage to discuss the plan with someone else in the anesthesia team, they choose not to, and indicated that this posed a challenge for safeguarding the patient. Despite not verbally voicing their plan, the RNAs had a mental plan, which sometimes could be taken over by the anesthesiologist. For example, an anesthesiologist might enter the OR and start to change the settings for the ventilator mode or the dosage of anesthetic drugs: “Sometimes this person leaves the OR after a few minutes, but then the plan is already ruined “(p. 3). It was also indicated that the anesthesiologists disrupted their plans by coming into the OR and taking over the performance of the extubation without asking the RNAs whether they had prepared any plan for the extubation. Here, the RNAs felt as though they were being taken over, losing focus and had to reconsider their plan to be able to safeguard the patient in the process of extubation.

## Discussion

The aim of this study was to obtain a deeper understanding of Registered Nurse Anesthetists’ main concerns and how they solve these in the process of extubation when caring for a patient in the anesthesia setting. The results showed that *Safeguarding the patient in a highly technological environment* were the RNAs’ main concern, which they solved by *maintaining adaptability*. The grounded theory, *Safeguarding the patient in the process of extubation,* emerged from the data in its analysis, and adds to our understanding of how the RNAs cope with the vulnerable situation of the extubation.

The RNAs in this study continually oscillate in their interactions with the patients, between being able to stay attentive and having to contend with frequent disturbances in the complex practice of the process of extubation. As such, it illuminates how the RNAs try to cope with these interruptions by building plans, preparing themselves by using their previous experiences, and by interacting with the patient. According to Göras et al. [1], to create safe care and manage the complexity in the OR, certain resources and preconditions, such as working experience and resilience, were important in order to be able to adapt to, as well as to expect, unexpected situations. In this study, resilience is evident in how the RNAs simultaneously cope with both facilitators and challenges by maintaining adaptability to achieve the goal of moving towards a safe extubation and resolving their main concern of safeguarding the patient. Also, this allows them to adapt a sense of moral resilience; that is, the ability to take good actions [24], in relation to which part of the process they are in. This is similar to the findings of Göras et al. [1], showing that to oscillate between being attentive, staying focused, and being interrupted affected the RNAs’ ability to safeguard the patient in the highly technological environment. It was indicated, though, that they were able to cope with these interruptions and still maintain adaptability and safeguard the patient. Weick et al. [25] found that resilience emerges over time when learning from situations characterized by having to manage risks. This was also described by Rönnberg et al. [12], who found that recognizable patterns play a key role in clinical competence and the use of intuition in the process of extubation. Along with Larsson and Holmström’s [26] findings, the RNAs in this study used their senses to obtain an overview of the situation and at the same time stay focused on a specific task by using professional competence. Tanner [27] states that, in clinical decision-making practices, both analytical and intuitive components are included, and these are similar to clinical judgement, where clinical judgment means interpreting patients’ needs, and, by drawing conclusions, the professional decides whether to take action, using or modifying approved methods in reaction to the patient’s response. This result aligns with an understanding of professional knowledge that Polanyi (1966) describes as tacit knowledge, which signifies an ability to recognize and act upon the basis of implicit practical experience in a particular context, without always being able to articulate this knowledge. Gut feelings were explained by the RNAs to be used as a basis for making the decision on when to extubate, which they interpreted as intuition, in line with how RNAs describe clinical intuition, which is combined with theoretical knowledge and experience in their decision-making practices in the process of extubation [12].

Although the most critical part of the extubation is the precise moment of the extubation, the process already starts at the beginning of anesthesia care. By maintaining adaptability, which represents the core category in this study, the RNAs meet the ethical demands and ethical obligation of caring for and taking responsibility for the patients. This involves the patient transferring the responsibility for themselves to another person [28]. For the RNAs in this study, this means taking that responsibility and meeting this ethical demand by safeguarding the patient in a highly technological environment, and taking responsibility for the life that is placed in their hands. According to Løgstrup [29], when you encounter another person, you are holding a part of that person’s life in your hands, meeting ethical obligations, and connecting with another person.

In this study, safeguarding the patient in a highly technological environment meant that the RNAs act upon experiences they have of earlier extubations, and, by maintaining adaptability and being attentive, they enabled themselves to be sensitive and assume an openness to each unique patient’s reactions. By interacting attentively, the RNAs used the patient’s reactions and responses to anesthetics and stimuli to guide them in their decision-making practices during the process of extubation. As described by Rönnberg et al. [12], when RNAs combine their clinical experience with the information they attentively gather about the patient, they created a relationship by connecting with the patient. This involved the RNAs being mentally present for the patient, similar to having a presence when caring for a patient in the anesthesia setting, as described by Karlsson et al. [30]. Buber [31] describes that being present and truly listening involves having a receptive presence. Moreover, the challenge in oscillating between extremes, such as being fully focused while constantly interrupted, contributed to the complexity in the process of extubation. By being flexible and maintaining adaptability, the RNAs manage this complexity.

In the complex environment of the OR, Karlsson et al. [30] found that it is a challenge for the RNAs to remain in control of technical equipment while keeping the patient safe. In this study, the RNAs’ attentive interactions, and continuous evaluation of the patient’s reactions, were indicated to be a way of listening to the patient, and, through this, the RNAs were guided in how to act when safeguarding the patient in the process of extubation. This interaction is a relationship between the RNA and the patient and is affected when the RNA’s attention is diverted to, for example, alarms from technical equipment [30]. Sharing the responsibility of the extubation with someone else in the anesthesia team enables the RNAs to maintain attentiveness and safeguard the patient by staying focused and calm. This signifies a presence involving RNAs placing their attention on the patient and for them to remain by the patient’s side and to safeguard them. Safeguarding is one attribute of patient advocacy [32], including how the nurses track medical errors and protect the patients from misconduct or incompetency from co-workers. The RNAs in this study were focused on safeguarding the patient and had a backup plan in case unexpected events occurred. Likewise, the RNA takes on the role of the patient’s advocate, keeping vigil and engaging with them in the anesthesia setting, as similarly described by Schreiber and Macdonald [2]. Sundqvist et al. [33] found that being the patient’s advocate is about protecting them from harm in a vulnerable position, speaking for them, and caring for them during perioperative care. By maintaining adaptability, the RNAs in our study coped with disturbances and managed to hold the line to safeguard the patient. This has also been described as an ethical obligation by Sundqvist et al. [33, 34] when respecting the patient’s integrity and taking responsibility for the life that is placed into their hands. Being the patient’s advocate when they are unable to have control themselves is included in the RNA’s responsibilities; however, acting as someone’s advocate may cause conflicts with other professionals when protecting the patient’s integrity and autonomy, as described by Abelsson [35].

The theory divulged by this study in safeguarding the patient in the process of extubation has been shown to be complex, and the RNAs’ main concern was to safeguard the patient in a highly technological environment; this they resolved by maintaining adaptability. Brien et al. [36] state that a culture of safety is necessary in the process of extubation and in the perioperative setting, and this has been shown to benefit from good leadership, promoting an understanding of each other, and an awareness of the context, knowledge and the benefits of effective communication skills. Dealing with uncertainty due to feelings of vulnerability and not being able to focus hindered the RNAs in safeguarding the patient. This uncertainty may involve the RNAs’ level of education, experience, and available resources, which Seifert [37] relates to ethics and which is important in promoting patient advocacy in perioperative care [34]. Another theory of safeguarding, described by Solbakken et al. [38], includes nurses having a clinical presence, securing patients’ voices, and maintaining a trustful relationship. Interacting with the patient in the stressful working environment of the OR, and being repeatedly interrupted, also affected the RNAs’ ability to stay focused in the process of extubation. Hanssen et al. [39] have describe that respect and patient safety, along with ethics and adopting a moral attitude, are perceived as being central non-technical skills that are integrated in nursing practice. Lindwall and von Post [40] suggest that reflecting upon caring and ethical issues may create a tolerant atmosphere, where ethical dilemmas can be discussed. Nilsson and Jaensson [6] found that anesthetic nursing includes keeping in touch with the patient, watching over the patient, and being one step ahead. This study explains anesthetic nursing further as safeguarding the patient in the process of extubation, to constantly oscillate between facilitators and challenges while still keeping the patient’s safety in focus, managing all interactions and safeguarding the patient in a highly technological environment, adding knew knowledge about anesthetic nursing.

### Methodological considerations and limitations

Given the severity of the potential complications related to the extubation in the anesthesia setting, and the limited knowledge relating to RNAs’ experiences, it is important to gain a better understanding of the RNAs’ main concerns regarding the process of extubation. Therefore, a classic grounded theory [17, 14] methodology was considered suitable for this study, focusing on the process of extubation to find the main concerns of the participants and how these are resolved. GT is appropriate in research that focuses on processes that occur over time, follows several stages, and has a beginning and an end point [14], to reveal repeating patterns in the data.

Although Glaser [14] advises against audio-recording interviews, arguing that the method is inefficient, generates irrelevant data, and detracts the researcher from focusing on the delimitation of categories [16], we chose to record the interviews to allow us to focus on the RNAs’ reflections while watching the video recordings, and to facilitate the identification of the most relevant data within the research team during analysis.

A limitation in this study was that the RNAs in data collection I were being video-recorded, which might have affected how they handled the extubation. However, they were asked to reflect upon their actions afterwards, and in data collection II, in-depth interviews were performed. Using different ways of gathering data is a strength of GT methodology [17].

To judge the quality of a GT, the criteria *fit*, *relevance*, *workability*, and *modifiability* should be discussed [17, 15]. *Fit* deals with how closely the concepts are related to the phenomena they represent, and patterns in the data [41]. The theory in this study is based on the data, quotations from RNAs are presented and the steps taken in the data analysis, including the act of constant comparison, have been described. The *Relevance* of a theory has to do with whether it describes what is most important to the participants [41]. This has been achieved by initially using an inductive approach and letting the concepts come from the data, and by being aware of and making every effort to restrain preconceptions. A potential limitation in this study is that both the first and fourth authors are RNAs. However, having knowledge about the culture of the OR and the RNAs role may also be beneficial. In addition, the data have been cross-validated by the other three authors, who have experiences of surgical, critical care, and psychiatric nursing, and this variety in perspective also strengthens the trustworthiness.

To achieve *workability*, the categories must be adapted to the data and consist of variation in determining how to resolve the main concern to which the theory is applied [41]. The categories identified in this study are derived from the data and have broad variation in content, including both facilitators and challenges. A limitation in this study is that none of the participants have been asked to confirm the relevance or workability of the theory. However, the third author, who is an RNA, and who was not involved in performing data collection, confirmed that the result mirrored the reality of the process of extubation. The other three authors had no experience of working as RNAs, which was also considered to be a strength in the cross-validation of the workability of the theory. Finally, the criteria *modifiability* is considered to be fulfilled, as the theory is considered to be able to be modified if new and relevant data are compared to existing data.

## Conclusion

The process of extubation is not linear; instead, it is a series of actions and tasks performed and determined due to the anesthetized patient’s condition, reactions, and the outcome of surgery. RNAs must manage this process safely, despite the challenges of dealing with the uncertainty of the patient’s responses, and the distractions and interruptions from other professionals. The RNAs oscillate between facilitators and challenges to be able to safeguard the patient in a highly technological environment. This oscillating differs between patients and RNAs, and with RNAs’ working experience. The RNAs resolve these difficulties in oscillating by maintaining adaptability, indicating the need for finding a balance between maintaining attentiveness on what is important to keep the patient safe in the process of extubation and all of the disturbances present in the OR. This needs to be taken into consideration in education and clinical practice, especially for those who have less working experience in the anesthesia setting. Highlighting the complexity of this process, and placing a focus on this critical moment, will allow patient safety to be increased. Despite having to function in a complex working environment with frequent distractions, the RNAs manage to safeguard the vulnerable patient in the process of extubation. By creating a relationship with the patient, and focusing on the patient beyond the monitors, the RNAs provide safe nursing care.

The extubation is a critical moment for the patient. Sharing this experience provides a greater understanding of the main concern in the process of the extubation and how to resolve it. The results can be used in education programs for specialist training in anesthesia, and this knowledge may ultimately improve patient safety.

## Data Availability

Data from this study is not allowed to be publically available since it could compromise the privacy of the participants from interviews or transcripts. Only persons involved in the research group are allowed to access data. This was also the information the participants were given prior their participation. Also this was approved by the Regional ethical board in Umeå.

## Abbreviations

GA: General Anesthesia
GT: Grounded Theory
OR: Operating Room
RNA: Registered Nurse Anesthetist

## Footnotes

1 We chose this word specifically for its ability to clearly describe the experiences of the RNAs: to oscillate means ‘To alternate *between* two states, opinions, principles, purposes, etc.; to vary or fluctuate alternately *between* two limits’ (*Oxford English Dictionary*, italics in original).
